# Molecular cytogenetic characterisation of *Elytrigia* ×*mucronata*, a natural hybrid of *E. intermedia* and *E. repens* (Triticeae, Poaceae)

**DOI:** 10.1186/s12870-019-1806-y

**Published:** 2019-05-31

**Authors:** Ladislava Paštová, Alexander Belyayev, Václav Mahelka

**Affiliations:** 1 Institute of Botany, Czech Academy of Sciences, Zámek 1, 252 43 Průhonice, Czech Republic; 2 Department of Botany, Charles University, Benátská 2, 128 01 Prague, Czech Republic

**Keywords:** Allopolyploidy, Chromosomal alterations, *Elymus repens*, Higher polyploids, Hybridisation, FISH, GISH, *Thinopyrum intermedium*

## Abstract

**Background:**

Interspecific hybridisation resulting in polyploidy is one of the major driving forces in plant evolution. Here, we present data from the molecular cytogenetic analysis of three cytotypes of *Elytrigia* ×*mucronata* using sequential fluorescence (5S rDNA, 18S rDNA and pSc119.2 probes) and genomic in situ hybridisation (four genomic probes of diploid taxa, i.e., *Aegilops*, *Dasypyrum*, *Hordeum* and *Pseudoroegneria*).

**Results:**

The concurrent presence of *Hordeum* (descended from *E. repens*) and *Dasypyrum* + *Aegilops* (descended from *E. intermedia*) chromosome sets in all cytotypes of *E.* ×*mucronata* confirmed the assumed hybrid origin of the analysed plants. The following different genomic constitutions were observed for *E.* ×*mucronata*. Hexaploid plants exhibited three chromosome sets from *Pseudoroegneria* and one chromosome set each from *Aegilops, Hordeum* and *Dasypyrum*. Heptaploid plants harboured the six chromosome sets of the hexaploid plants and an additional *Pseudoroegneria* chromosome set. Nonaploid cytotypes differed in their genomic constitutions, reflecting different origins through the fusion of reduced and unreduced gametes. The hybridisation patterns of repetitive sequences (5S rDNA, 18S rDNA, and pSc119.2) in *E. ×mucronata* varied between and within cytotypes. Chromosome alterations that were not identified in the parental species were found in both heptaploid and some nonaploid plants.

**Conclusions:**

The results confirmed that both homoploid hybridisation and heteroploid hybridisation that lead to the coexistence of four different haplomes within single hybrid genomes occur in *Elytrigia* allopolyploids. The chromosomal alterations observed in both heptaploid and some nonaploid plants indicated that genome restructuring occurs during and/or after the hybrids arose. Moreover, a specific chromosomal translocation detected in one of the nonaploids indicated that it was not a primary hybrid. Therefore, at least some of the hybrids are fertile. Hybridisation in *Triticeae* allopolyploids clearly and significantly contributes to genomic diversity. Different combinations of parental haplomes coupled with chromosomal alterations may result in the establishment of unique lineages, thus providing raw material for selection.

**Electronic supplementary material:**

The online version of this article (10.1186/s12870-019-1806-y) contains supplementary material, which is available to authorized users.

## Background

Hybridisation and polyploidisation are the major driving forces underlying plant evolution [1–4]. While hybridisation through genome merging may lead to the formation of new hybrid species, polyploidisation can stabilise hybrid breeding behaviour [5].

Hybridisation can occur between species of the same ploidy level (homoploid hybridisation) and between species of different ploidy levels (heteroploid hybridisation). In either case, the formation of new hybridogenous species in sympatry requires the presence of reproductive barriers between the hybrid and its parents. In the absence of reproductive barriers, newly formed hybrids can backcross with one or both parental species and form hybrid swarms [6].

The newly formed hybrid possesses a novel combination of genomes, which can manifest improved or enhanced qualities for certain characteristics (hybrid vigour or heterosis). However, hybrid vigour decreases in every subsequent generation of a hybrid’s progeny because of the decreasing proportion of heterozygotes.

Allopolyploidisation, i.e., the multiplication of chromosome sets in hybrids, is a mechanism by which the decay of hybrid vigour can be avoided. In allopolyploids, the advantage of heterosis is largely retained due to the enforced pairing of homologous chromosomes and limited intergenomic recombination [1].

The involvement of unreduced gametes in mating is considered to be the prevailing mechanism for the origin of polyploids [7]. Alternative polyploid formation mechanisms include polyspermy (fertilisation by more male gametes; [8]) and (somatic) genome doubling [9]. Thus, the established allopolyploids can generate high ploidy level cytotypes in polyploid complexes.

The changes that occur after polyploid hybrid formation include diverse processes at the molecular, chromosomal, and organismal levels [10]. Coexistence of formerly isolated genomes is often followed by chromosomal restructuring, which contributes to genome variation beyond the merging of genomes [11].

The tribe *Triticeae* is an extensively studied group of grasses in which hybridisation and polyploidisation have significantly contributed to present-day diversity. Depending on the classification, *Triticeae* comprises approximately 350–500 taxa in 27–37 genera [12–14]. In total, 23 basic genomes were distinguished by Löve, who referred to them to as haplomes [13].

The basic chromosome number in *Triticeae* is *x* = 7. The majority of species are allopolyploids, with ploidy levels ranging from tetraploid (2n = 4x) to dodecaploid (2n = 12x). The structure of the tribe is reticulate when certain haplomes are involved in the formation of more than one allopolyploid species [13].

*Elytrigia* ×*mucronata* (Opiz) Prokudin [syn. *Agropyron mucronatum* Opiz, *E. apiculata* (Tscherning) Jirásek], the subject of this study, is a natural hybrid between *E. intermedia* (Host) Nevski [syn. *Elymus hispidus* (Opiz) Melderis, *Thinopyrum intermedium* (Host) Barkworth & D.R. Dewey] and *E. repens* (L.) Nevski [syn. *Elymus repens* (L.) Gould]. Both parental species occur predominantly as hexaploids (2n = 6x = 42) in the study area (Czech Republic), but a minority nonaploid cytotype (2n = 9x = 63) was recorded for *E. repens* [15, 16]. Natural hybridisation between the hexaploids *E. intermedia* and *E. repens* appears to be common and is bi-directional, and evidence of backcrosses has been found [16]. Therefore, no obvious reproductive barriers exist in this species complex. In addition to the prevailing hexaploid *E.* ×*mucronata*, hybrids with higher ploidy occur rarely, including heptaploids (2n = 7x = 49) in one population and nonaploids in three populations. The origin of the nonaploid hybrids has been assumed to have involved the fusion of unreduced and reduced gametes, where the unreduced gamete was donated by either of the parental species or their hybrid [16]. The origin of the heptaploids is unknown.

Both parental species are perennial allopolyploids with complex evolutionary histories [17–20]. The composition of the hexaploid *Elytrigia repens* genome is assumed to include two subgenomes originating from *Pseudoroegneria* (haplome symbol St) and one subgenome originating from *Hordeum* (H) (genomic formula StStStStHH). In addition to the major genome constituents, the *E. repens* genome contains hints of other lineages from within and outside of *Triticeae*, demonstrating that the species must have acquired additional genetic material from distant sources [17, 18, 21]. A portion of the foreign genetic material found in *E. repens* has been inherited from its parental species [22].

The genomic constitution of the allohexaploid intermediate wheatgrass *E. intermedia* has not yet been satisfactorily resolved (for discussion, see [20, 23]). A consensus has been reached that the species is composed of three distinct subgenomes, one of which originated from *Pseudoroegneria* (St). The identity of the other two subgenomes remains controversial, particularly due to assumed contributions from *Thinopyrum bessarabicum* (E^b^), *Th. elongatum* (E^e^), *Dasypyrum* (V), *Secale* (R), and *Aegilops* (D) [19, 20, 23, 24]. In this respect, the presence of potential local varieties cannot be entirely excluded. Nevertheless, data from the analysis of native Central European specimens from the Czech Republic (from the same area from which the studied hybrids originated) clearly suggest contributions from *Dasypyrum* (V) and *Aegilops* (D) [19, 23]. Therefore, we concluded that these genera represent the donors of the two other subgenomes in this allopolyploid, and we therefore assume the genomic formula of *E. intermedia* to be StStDDVV.

Molecular cytogenetics techniques have given rise to new possibilities for studying the genomic constitution of hybrid plants. In particular, genomic in situ hybridisation (GISH) is suitable for studying the origins of allopolyploid species (e.g., [18, 25]). Moreover, both numerical and structural chromosomal alterations (especially intergenomic translocations) can be detected in allopolyploids [26, 27]. Furthermore, fluorescence in situ hybridisation (FISH) employing known repetitive sequences as chromosomal markers provides information on their physical localisation within the genome and allows us to study genome restructuring after polyploidisation events [28, 29].

In this study, we performed molecular cytogenetic analyses of three cytotypes of the allopolyploid hybrid *E.* ×*mucronata* to characterise their genomic constitutions and elucidate their genome dynamics following hybrid formation. In particular, sequential GISH and FISH were employed to reveal (1) the genomic constitution of the hexaploid, heptaploid, and nonaploid cytotypes of *E.* ×*mucronata*; (2) chromosomal alterations in the studied genotypes; and (3) the patterns of the ribosomal 5S and 18S rDNA units with the *Triticeae*-specific repeat pSc119.2, primarily with respect to their assignment to particular haplomes.

## Results

**Fig. 1:**
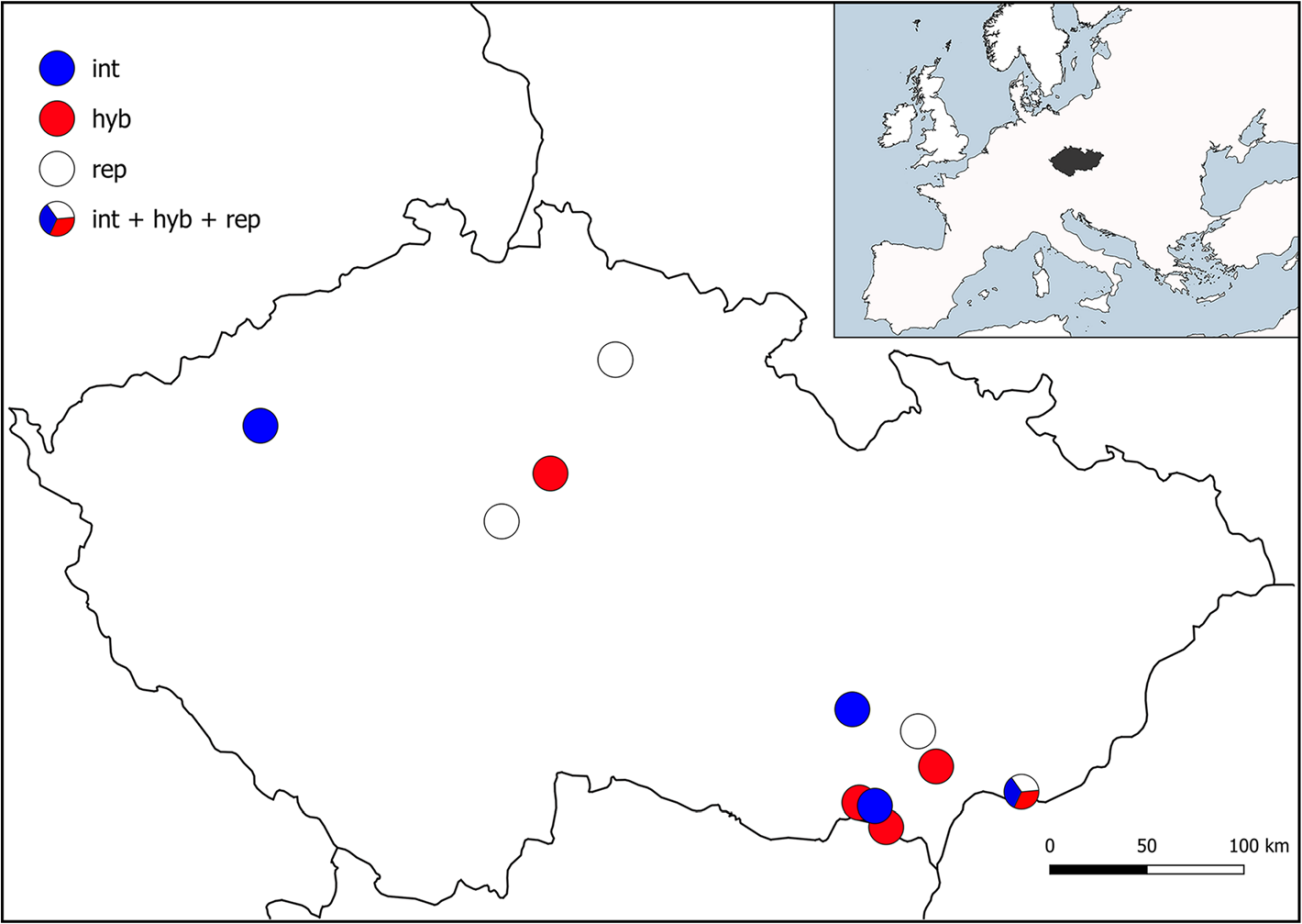
Map showing the location of the studied plants of *E. ×mucronata*. As complementary information, the locations of the parental species analysed in other studies (see text) is given. Blue circle (int) – *Elytrigia intermedia*; red circle (hyb) – *E. ×mucronata;* empty circle (rep) – *E. repens*; combined circle (int + hyb + rep) – all three taxa originated from the locality

Plant material for this study is available in previous studies [15, 16]. The experiments were performed on two hexaploid (2n = 6x = 42), two heptaploid (2n = 7x = 49), and three nonaploid (2n = 9x = 63) *Elytrigia ×mucronata* plants from five Central European localities (Table 1, Fig. 1). While the hexaploid and nonaploid plants were used in previous studies focused on genome size variation and natural hybridisation ([15, 16]; for details see Methods Plant material), the heptaploids were studied for the first time in this paper. The plants were identified based on morphological, flow cytometric, and ITS diagnostic markers [15, 16].

**Table 1:** List of the localities of the analysed plants

Locality (location, habitat)	Coordinates	Ploidy levels/plant ID		
		6x	7x	9x
1. Paví vrch (2 km S of Sedlec village, Paví vrch hill, steppe and field margin)	48°45′50.8″ N 16°41′33.1″ E	10–1		
2. Vrbčany (1.5 km NE of Vrbčany village, steppic slope)	50°03′43.5″ N 14°59′56.0″ E	17–4		
3. Čertoryje (4 km SE of Tvarožná Lhota village, Čertoryje National Nature Reserve, mesophilous meadow)	48°51′34.6″ N 17°24′32.0″ E		C9, C25B	
4. Hovorany (1 km NW of Hovorany village, Hovoranské louky Nature Reserve, steppic grassland)	48°57′54.2″ N 16°58′25.7″ E			41–5
5. Dolní Dunajovice (0.8 km W of Dolní Dunajovice village, field margin)	48°51′18.1″ N 16°33′34.9″ E			50–1, 50–7

Geographic location, habitat type and coordinates are given. All plants were collected in the Czech Republic

### Hexaploid Elytrigia ×mucronata

#### Genome composition

**Fig. 2:**
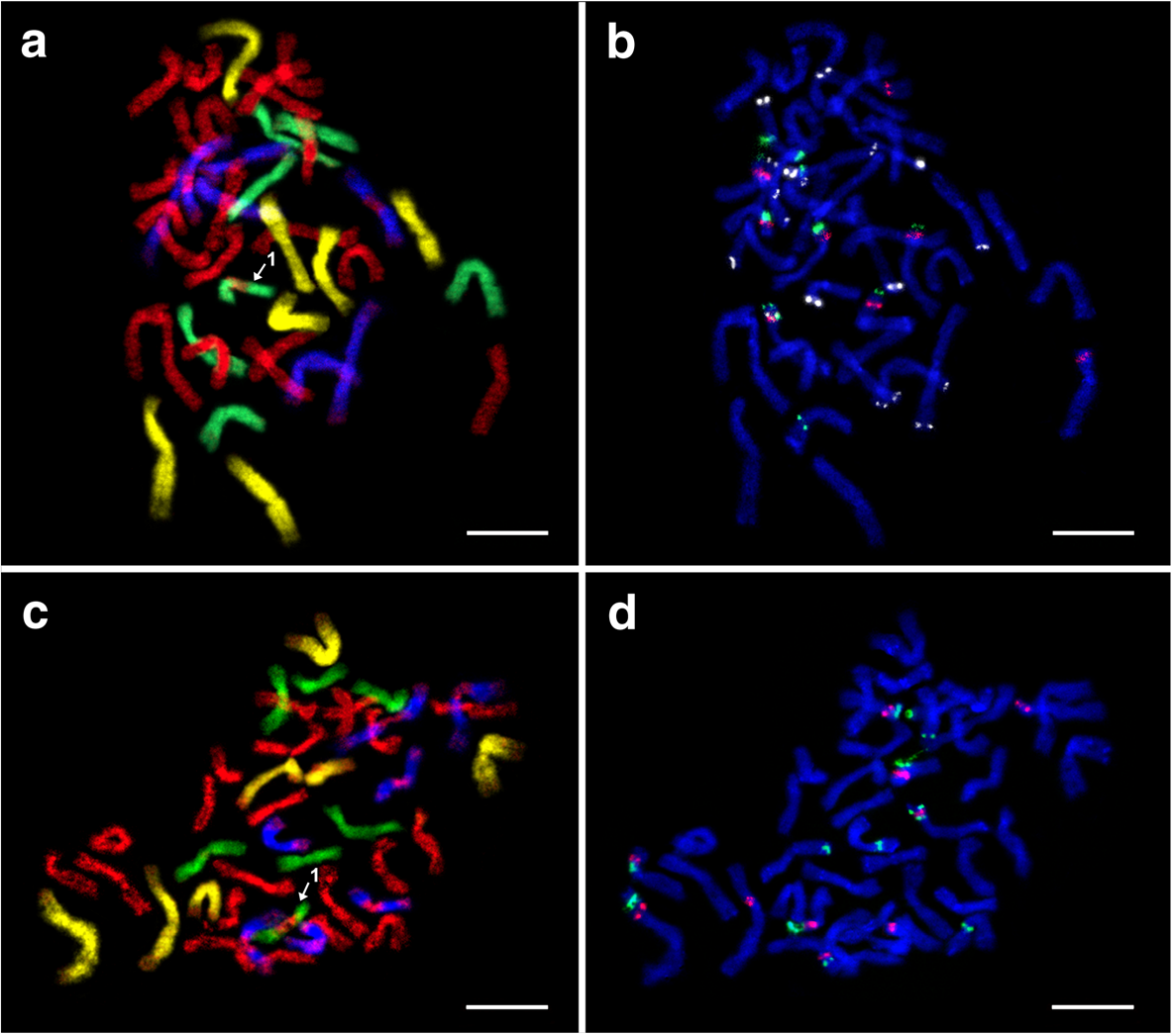
Mitotic metaphase chromosomes of hexaploid *E. ×mucronata* after sequential FISH and GISH experiments*.* **a** and **c** GISH in plants 10–1 and 17–4: *Aegilops tauschii* (digoxigenin, anti-DIG-FITC, yellow pseudocolour), *Dasypyrum villosum* (biotin, streptavidin-Cy3, blue pseudocolour)*, Hordeum bogdanii* (biotin, streptavidin-Cy3, green pseudocolour), and *Pseudoroegneria spicata* (Cy5, red). **b** and **d** FISH in plants 10–1 and 17–4: 5S rDNA (Cy5, red), 18S rDNA (digoxigenin, anti-DIG-FITC, green), and pSc119.2 (Cy3, white pseudocolour; applied only in 10–1 – b). After FISH, chromosomes were counterstained with DAPI. Structural chromosomal alterations are indicated by numerals (1). Scale bars = 10 μm

**Fig. 3:**
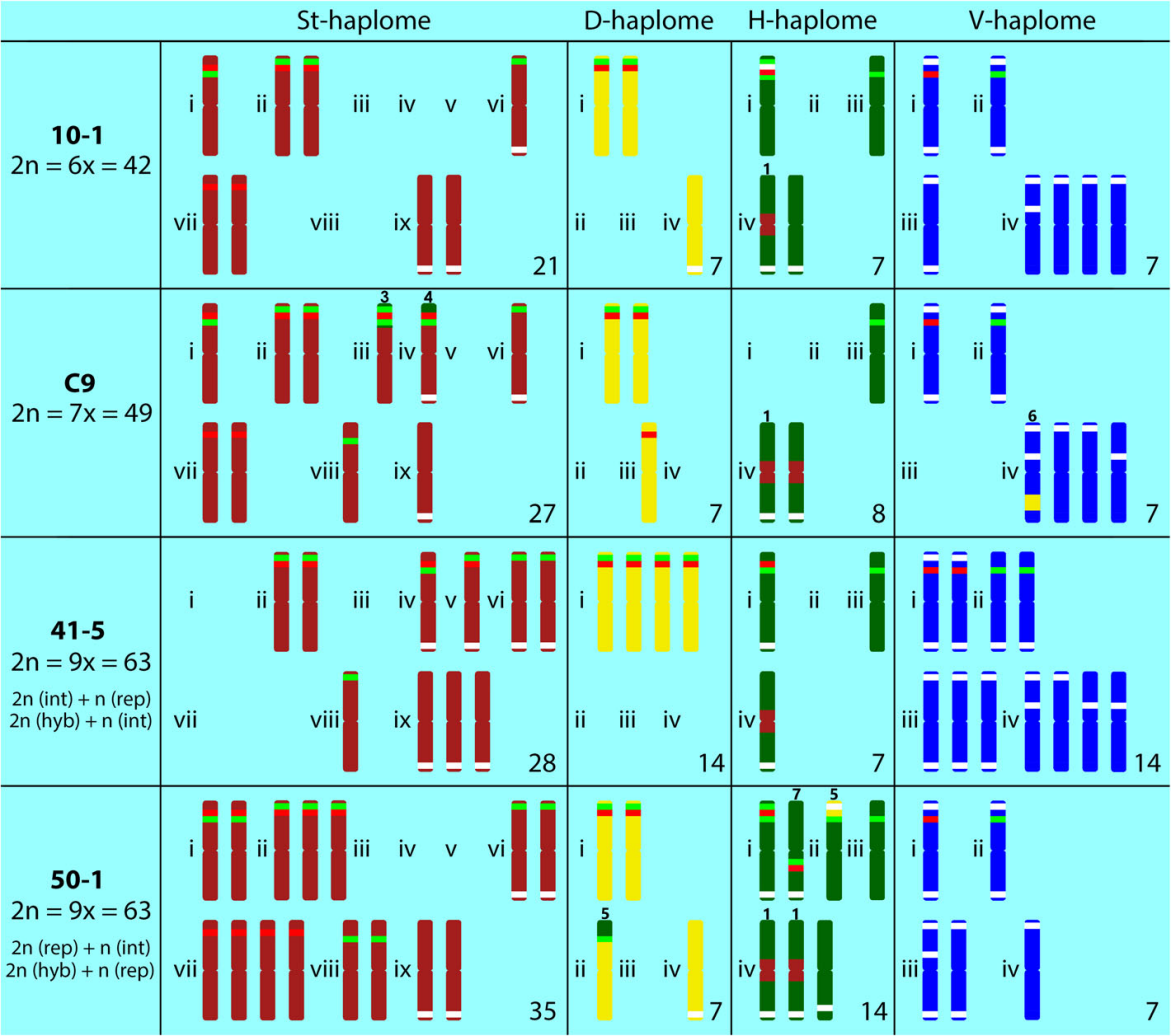
Schematic patterns of rDNA and pSc119.2 sites revealed in three cytotypes of *E. ×mucronata.* Only chromosomes exhibiting a signal after FISH are shown. Chromosomes with identical/similar patterns within each haplome are arranged into groups indicated with roman numerals. St haplome – dark red, D haplome – yellow, H haplome – green, V haplome – blue, 5S rDNA – red, 18S rDNA – light green, and pSc119.2 – white. Different types of structural chromosomal alterations are indicated by numerals above the chromosomes (1–7). Total numbers of chromosomes of a particular haplome are given (bottom right side). One *Pseudoroegneria* chromosome was missing in the analysed metaphase of 41–5

We analysed two hexaploid (2n = 6x = 42) *E*. ×*mucronata* plants. The plants 10–1 and 17–4 originated from localities ‘Paví vrch’ and ‘Vrbčany’, respectively, (Table 1). After GISH, we observed identical hybridisation patterns in both analysed plants, which consisted of 21 St (*Pseudoroegneria*) + 7 H (*Hordeum*) + 7 D (*Aegilops*) + 7 V (*Dasypyrum*) chromosomes (Fig. 2a and c; Table 2). A signal was missing in the centromeric regions of *Dasypyrum*-labelled chromosomes. We are currently not convinced that this pattern is an indication of chromosomal translocations. In contrast, only one of the *Hordeum*-labelled chromosomes exhibited a *Pseudoroegneria* probe signal in the centromeric region, suggesting the presence of an intergenomic translocation (Figs. 2a, c and 3).

**Table 2:** Number of rDNA and pSc119.2 sites in *E. ×mucronata*

Ploidy level/plant ID		No. of chromosomes of particular haplomes	Total no. of rDNA loci		No. of co-localised rDNA loci			Total no. sites/chromosomes
			5S	18S	5S–18S^1^	18S-5S^2^	18S–5S-18S^3^	pSc119.2
2n = 6x = 42								
10–1			9	10	1	4	1	18/14
17–4			10	13	1	5	1	–
10–1	St	21	5	4	1	2	0	3/3
	D	7	2	2	0	2	0	1/1
	H	7	1	3	0	0	1	3/3
	V	7	1	1	0	0	0	11/7
17–4	St	21	5	7	1	3	0	–
	D	7	3	2	0	2	0	–
	H	7	1	3	0	0	1	–
	V	7	1	1	0	0	0	–
2n = 7x = 49								
C25B			12	13	2	5	1	–
C9			11	12	2	4	1	14/11
C25B	St	28	8 (+ 1)	9 (+ 2)	2	3	1 (+ 1)	–
	D	7	3	2	0	2	0	–
	H	7	0 (−1)	1 (−2)	0	0	0 (−1)	–
	V	7	1	1	0	0	0	–
C9	St	27^A-1^	7 (+ 1)	8 (+ 2)	2	2	1 (+ 1)	3/3
	D	7	3	2	0	2	0	0
	H	8^A + 1^	0 (−1)	1 (−2)	0	0	0 (−1)	2/2
	V	7	1	1	0	0	0	9/6
2n = 9x = 63								
50–1			14	18	3	5	1	21/16
50–7			11	16	3	4	1	20/15
41–5			11	15	2	7	0	26/20
50–1	St	35	9	9	2	3	0	4/4
	D	7	2	3	0	2	0	1(−1)/1
	H	14	2	5	1	0	1	6(+ 1)/6
	V	7	1	1	0	0	0	10/5
50–7	St	35	5	8	2	2	0	3/3
	D	7	3	2	0	2	0	2/2
	H	14	2	5	1	0	1	5/5
	V	7	1	1	0	0	0	10/5
41–5	St	28	4	7	1	3	0	7/7
	D	14	4	4	0	4	0	0
	H	7	1	2	1	0	0	2/2
	V	14	2	2	0	0	0	17/11

^1^18S rDNA is situated proximal to the 5S rDNA locus (the mutual position of the 5S and 18S rDNA loci on the chromosome in the direction from the centromere to the telomere)

^2^18S rDNA is situated distal to the 5S rDNA locus

^3^18S rDNA is situated both distal and proximal to the 5S rDNA locus

^A+1^aneuploid chromosome set – one extra chromosome

^A-1^aneuploid chromosome set – one missing chromosome

(+numeral) number of additional loci acquired by a translocated segment from another haplome

(–numeral) number of loci translocated to another haplome

#### Chromosomal structural variation

In the plant 10–1, in which the complete set of FISH probes was applied, 5S rDNA, 18S rDNA and pSc119.2 signals were located on all of the chromosome sets (haplomes; Figs. 2b and 3; Table 2), with 9, 10 and 18 probe hybridisation sites being observed in this plant, respectively. *Pseudoroegneria*-labelled chromosomes (St haplome) carried five 5S and four 18S rDNA sites. The 5S rDNA co-localised with 18S rDNA on three chromosomes; on two of these chromosomes, the 5S rDNA loci were situated proximal to 18S rDNA, and on one of them, the 5S rDNA locus was located distal to the 18S rDNA. Additionally, there were two chromosomes carrying solitary subtelomeric 5S rDNA loci and a single chromosome carrying a subtelomeric 18S rDNA locus. Within the *Aegilops*-labelled chromosomes (D haplome), we detected two chromosomes with co-localised 5S and 18S rDNA loci, where the latter was positioned distal to the former. The *Hordeum*-labelled chromosomes (H haplome) carried one 5S rDNA site and three 18S rDNA sites. On one of the chromosomes, there was a co-localised rDNA locus containing a double 18S rDNA site with an intervening 5S rDNA site (18S–5S–18S). The V haplome, corresponding to *Dasypyrum*, carried single 5S and 18S rDNA sites located on separate chromosomes.

FISH with pSc119.2 revealed a disproportion compared to the number of detected sites (Fig. 2b; Table 2). The *Pseudoroegneria*-labelled chromosomes carried three pSc119.2 sites, one of which was located on the chromosome carrying the single 18S rDNA locus on the opposite arm. A single pSc119.2 hybridisation site in the terminal region was detected within the *Aegilops*-labelled chromosomes. The H haplome from *Hordeum* carried three pSc119.2 loci. Two of these loci resided on chromosomes lacking rDNA sites, while the third was located within the co-localised rDNA locus. The seven *Dasypyrum*-labelled chromosomes carried eleven pSc119.2 hybridisation sites in total. Three of these chromosomes harboured pSc119.2 in both terminal chromosome regions, one chromosome carried two loci on the same chromosome arm; the other chromosomes carried only one site at one chromosome end.

A slightly different pattern was observed in the second hexaploid plant (17–4) upon analyses with FISH with rDNA probes. There were three additional 18S rDNA sites on the *Pseudoroegneria*-labelled chromosomes and an additional 5S rDNA site on the *Aegilops*-labelled chromosomes (Fig. 2c and d; Table 2). The obtained results convincingly showed that the hexaploid plants are hybrids between hexaploid *E. intermedia* and *E. repens*.

### Heptaploid Elytrigia ×mucronata

#### Genome composition

**Fig. 4:**
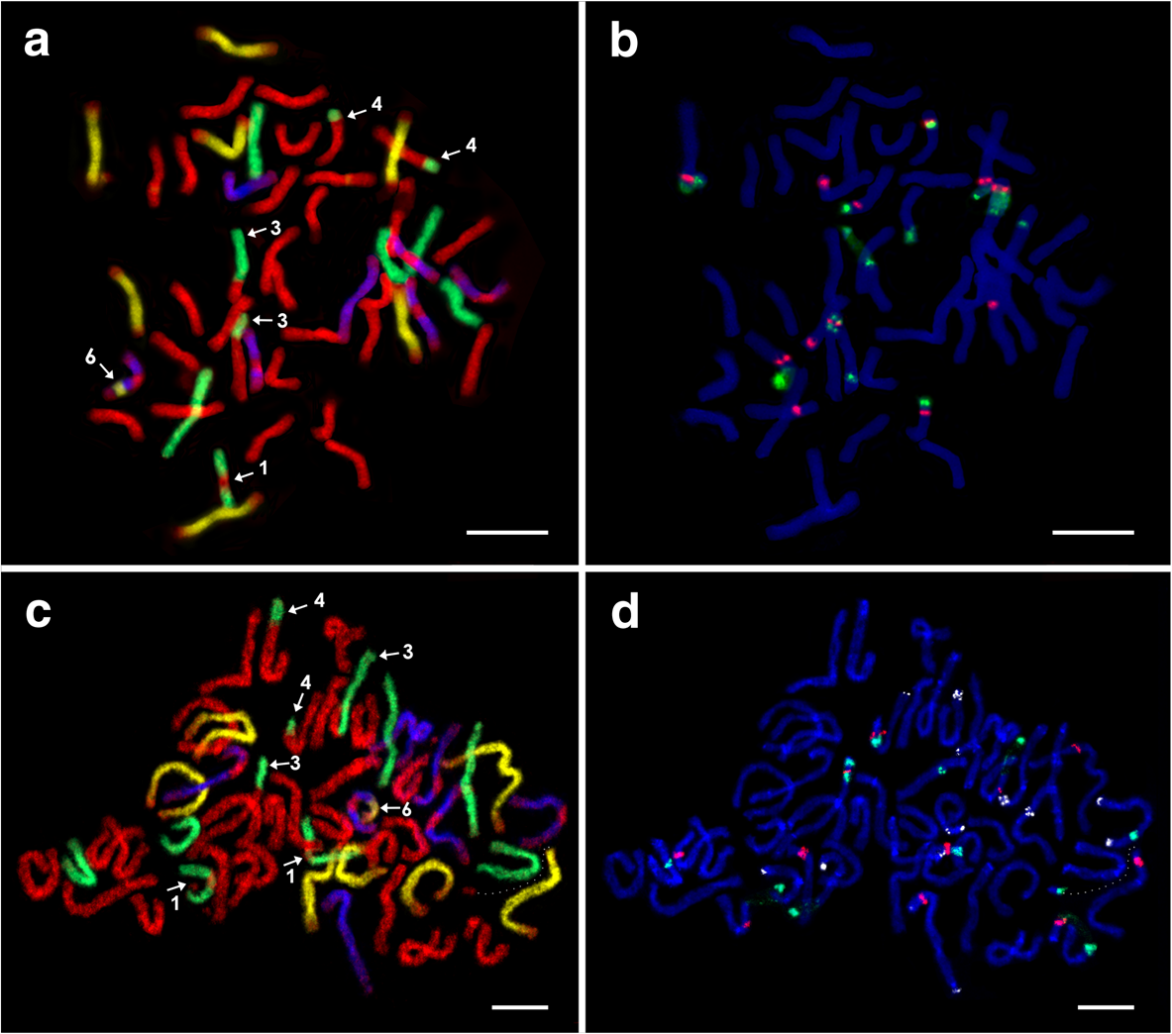
Mitotic metaphase chromosomes of heptaploid *E. ×mucronata* after sequential FISH and GISH experiments*.* **a** and **c** GISH in plants C25B and C9: *Aegilops tauschii* (digoxigenin, anti-DIG-FITC, yellow pseudocolour), *Dasypyrum villosum* (biotin, streptavidin-Cy3, blue pseudocolour)*, Hordeum bogdanii* (biotin, streptavidin-Cy3, green pseudocolour), and *Pseudoroegneria spicata* (Cy5, red). **b** and **d** FISH in plants C25B and C9: 5S rDNA (Cy5, red), 18S rDNA (digoxigenin, anti-DIG-FITC, green), and pSc119.2 (Cy3, white pseudocolour – applied only in C9 – d). After FISH, chromosomes were counterstained with DAPI. Different types of structural chromosomal alterations are indicated with numerals (1, 3, 4, 6). The chromosomal segment broken from the *Aegilops* chromosome is connected by a dotted line (c and d). Scale bars = 10 μm

The two heptaploid (2n = 7x = 49) *E*. ×*mucronata* plants C9 and C25B, both from the locality ‘Čertoryje’ (Table 1), were analysed. The probe hybridisation patterns after GISH slightly differed between the two examined heptaploids. In addition, 28 St + 7 H + 7 D + 7 V chromosomes were found in plant C25B (Fig. 4a), whereas plant C9 exhibited 27 St + 8 H + 7 D + 7 V chromosomes (Fig. 4c). Similar to the hexaploids, five of the *Dasypyrum*-labelled chromosomes lacked a probe signal in centromeric regions. Several translocations involving all four haplomes were detected in both examined plants. The translocations are described in a separate chapter (see below).

#### Chromosomal structural variation

The contribution of additional chromosomes compared to the hexaploids is reflected by the increased numbers and altered patterns of the chromosomes marked by repetitive probes. There were differences between the two examined heptaploids regarding the *Pseudoroegneria* and *Hordeum* chromosome sets. The patterns within the other two haplomes were identical in both plants (although pSc119.2 was not applied in C25B).

The heptaploid C25B, which carries euploid chromosome sets for each haplome, exhibited twelve and thirteen 5S and 18S rDNA sites, respectively (Fig. 4b; Table 2). The heptaploid C9 exhibited eleven 5S and twelve 18S rDNA sites (Fig. 4d; Table 2). The differences between the two examined heptaploids are due to the acquisition of one extra *Hordeum*-like chromosome and the loss of one *Pseudoroegneria-*like chromosome in C9. Thus, in the C25B plant, the *Pseudoroegneria* chromosomes harboured eight 5S sites and nine 18S rDNA sites, while seven and eight corresponding sites were found in the C9 plant. The number of pSc119.2 sites on the *Pseudoroegneria* chromosomes in C9 remained the same as in the hexaploid (3 sites on 3 chromosomes). Two chromosomes exhibited the same pSc119.2 pattern observed in the hexaploid. The third chromosome, which displayed a pSc119.2 site on one chromosome arm, also exhibited a co-localised rDNA locus adjacent to the translocated terminal segment from a *Hordeum* chromosome on the opposite chromosome arm (Figs. 3 and 4c and d; Table 2).

The D haplome from *Aegilops* exhibited two chromosomes with co-localised 5S and 18S rDNA sites (5S situated proximally); moreover, there was an additional 5S site in the terminal region of one chromosome. No pSc119.2 sites were detected in the D haplome (in plant C9). The co-localised 18S–5S-18S locus, which was observed within the *Hordeum* haplome in hexaploids, was translocated to a *Pseudoroegneria*-like chromosome. Thus, the *Hordeum* haplome of the heptaploids carried only a single 18S rDNA site. However, the pSc119.2 locus residing within the original 18S–5S–18S rDNA locus in *Hordeum* was no longer detected within the translocated locus in *Pseudoroegneria*. The *Dasypyrum-*like chromosome set (in plant C9) harboured the same number of rDNA loci found in the hexaploids (one 5S and one 18S rDNA locus on separate chromosomes) but exhibited only nine pSc119.2 sites on six chromosomes (Figs. 3 and 4d, Table 2).

### Nonaploid Elytrigia ×mucronata

#### Genome composition

Three nonaploid (2n = 9x = 63) *E*. ×*mucronata* plants from two localities were analysed (Table 1). We chose plants with two different assumed origin scenarios for the analyses. While plants 50–1 and 50–7 from locality ‘Dolní Dunajovice’ may have arisen from a 2n (*E. repens*) + n (*E. intermedia*) or 2n (6x *E*. ×*mucronata*) + n (*E. repens*) combination, the nonaploid 41–5 from locality ‘Hovorany’ may be characterised by either a 2n (*E. intermedia*) + n (*E. repens*) or 2n (6x *E*. ×*mucronata*) + n (*E. intermedia*) gamete composition [16].

**Fig. 5:**
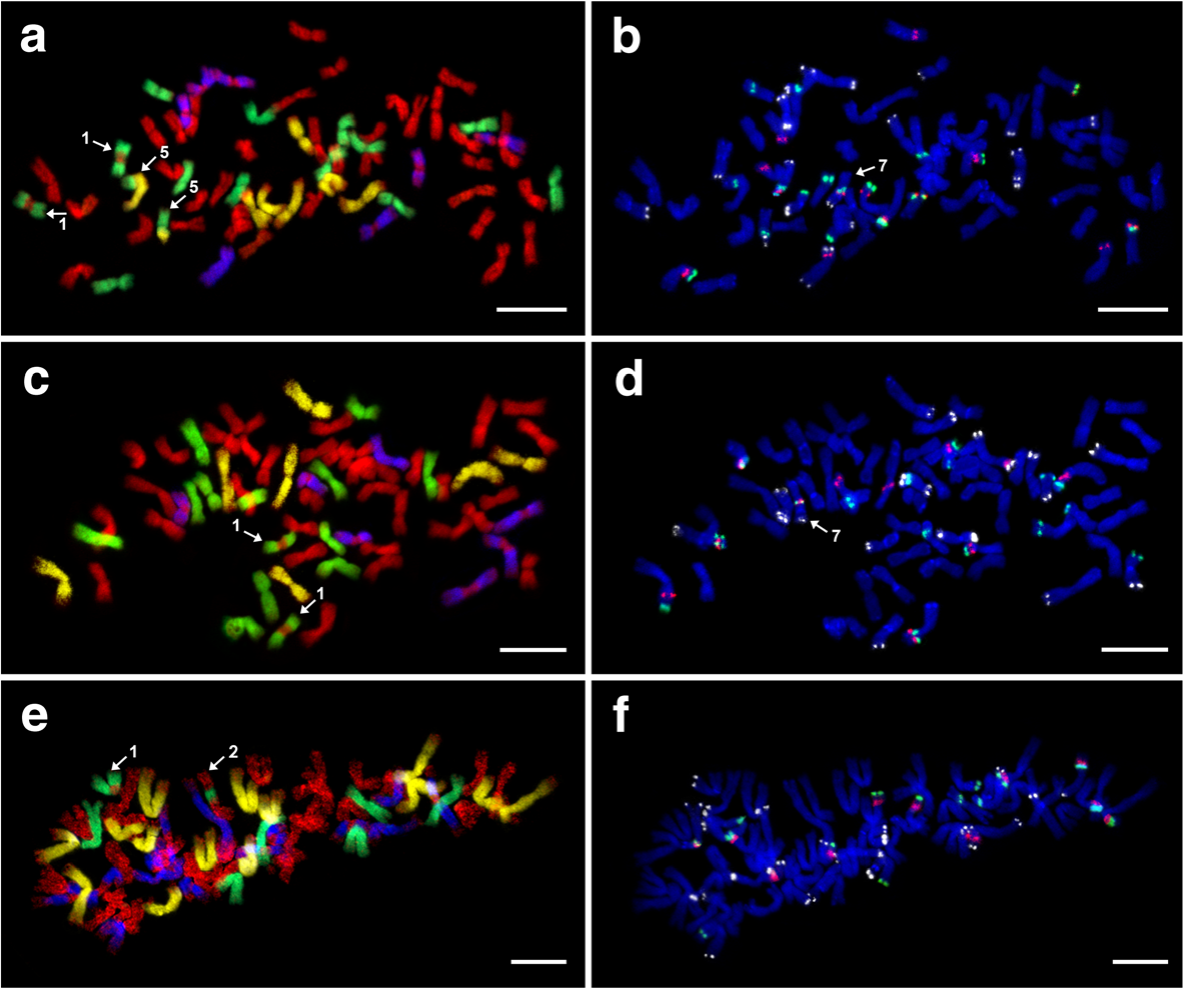
Mitotic metaphase chromosomes of nonaploid *E. ×mucronata* after sequential FISH and GISH experiments. **a**, **c**. and **e** GISH in plants 50–1, 50–7 and 41–5 (metaphase only with 62 chromosomes): *Aegilops tauschii* (digoxigenin, anti-DIG-FITC, yellow pseudocolour), *Dasypyrum villosum* (biotin, streptavidin-Cy3, blue pseudocolour)*, Hordeum bogdanii* (biotin-streptavidin-Cy3, green pseudocolour), and *Pseudoroegneria spicata* (Cy5, red). **b**, **d** and **f** FISH in plants 50–1, 50–7 and 41–5 (metaphase only with 62 chromosomes): 5S rDNA (Cy5, red), 18S rDNA (digoxigenin, anti-DIG-FITC, green), and pSc119.2 (Cy3, white pseudocolour). After FISH, chromosomes are counterstained with DAPI. Different types of structural chromosomal alterations were indicated with numerals (1, 2, 5, 7). Scale bars = 10 μm

There were two clear-cut GISH patterns among the analysed nonaploids, which likely reflected their distinct origins (see also Discussion). Two plants from one population (50–1 and 50–7) exhibited a 35 St + 14 H + 7 D + 7 V constitution (Fig. 5a and c). We again encountered a lack of signal from the *Dasypyrum* probe in the centromeric regions of *Dasypyrum*-labelled chromosomes. Chromosomal translocations were detected in both plants (for details, see below).

The genomic constitution of the 41–5 plant was different from that of the above-described plants (Fig. 5e). Based on the GISH analysis, 41–5 exhibited 28 St + 7 H + 14 D + 14 V chromosomes. Similar to the other analysed plants, the *Dasypyrum*-labelled chromosomes lacked a signal in centromeric regions.

#### Chromosomal structural variation

The examined nonaploid plants showed variable patterns in the FISH analysis, reflecting their distinct origins and genome additivity (50–1 and 50–7 vs. 41–5). Furthermore, differences in the FISH patterns were observed between two plants with an identical genomic constitution (50–1 and 50–7). In this case, the differences were mainly due to polymorphisms within the chromosome sets originating from *Pseudoroegneria*. Compared with the hexaploids, there was an increase in the total number of all FISH probes in all examined nonaploids (Table 2).

Plant 50–1 harboured a total of fourteen 5S and eighteen 18S rDNA sites. The pSc119.2 probe hybridised to 21 sites on 16 chromosomes. Five chromosome sets from the *Pseudoroegneria* St haplome exhibited nine 5S and nine 18S rDNA sites. These rDNA sites co-localised on five chromosomes, while four chromosomes harboured solitary 5S rDNA sites, and four other chromosomes harboured solitary 18S rDNA sites (two interstitial and two subtelomeric sites). The St haplome carried four pSc119.2 loci in the terminal chromosome region, two of which resided on 18S rDNA-carrying chromosomes on opposite chromosome arms. The *Aegilops*-like D haplome carried two 5S rDNA sites, three 18S rDNA sites and one pSc119.2 site. Two 5S and five 18S rDNA sites were detected within the two *Hordeum* chromosome sets. The 5S and 18S rDNA loci co-localised on two chromosomes. In addition to the 18S–5S–18S rDNA locus observed in both the hexaploids and heptaploids, there was an additional co-localised 5S–18S rDNA locus residing on the same chromosome arm as the pSc119.2 site. In total, six pSc119.2 sites were located on six *Hordeum* chromosomes. One of the sites resided on the terminal chromosome segment originating from an *Aegilops*-like chromosome. Similar to the hexaploids, the V haplome from *Dasypyrum* harboured solitary 5S and 18S rDNA sites located on separate chromosomes. Five out of seven *Dasypyrum-*like chromosomes carried ten pSc119.2 loci (Figs. 3 and 5b; Table 2).

Plant 50–7 harboured eleven 5S and sixteen 18S rDNA sites. The pSc119.2 probe hybridised at 20 sites on 15 chromosomes. The FISH pattern was similar to that of plant 50–1, with major differences present within the *Pseudoroegneria*-like chromosomes. These chromosome sets carried five 5S sites and eight 18S rDNA sites. Furthermore, the *Aegilops*-like chromosomes bore three 5S sites instead of two; two 18S rDNA loci instead of three; and two pSc119.2 hybridisation sites. The rDNA-FISH patterns within the haplomes from *Hordeum* and *Dasypyrum* were otherwise identical in the two plants. Five pSc119.2 loci were found on five *Hordeum-*labelled chromosomes*.* The number and pattern of pSc119.2 loci were the same in the *Dasypyrum* chromosome set (Fig. 5d; Table 2).

For plant 41–5, 28 *Pseudoroegneria*-labelled chromosomes carried four 5S and seven 18S rDNA sites. All of the 5S rDNA sites co-localised with 18S rDNA sites, and three of them were located proximal to 18S rDNA. The other 18S rDNA sites resided on three chromosomes in terminal regions. There were seven pSc119.2 loci located in terminal regions, four of which were located on rDNA-carrying chromosomes.

The Hordeum haplome of the nonaploid 41–5 carried one co-localised locus with a proximal 18S site and a distal 5S rDNA site, residing on the opposite chromosome arm to the pSc119.2 site. Moreover, there was a single 18S locus on a separate chromosome. The rDNA loci in both the D and V haplomes exhibited twice the number of sites found within these haplomes in the hexaploids. The D haplome carried two pairs of chromosomes with co-localised 5S and 18S rDNA sites, and the V haplome from *Dasypyrum* carried two 5S and two 18S sites, all of which resided on separate chromosomes. While the V haplome harboured seventeen pSc119.2 sites on 11 chromosomes, no pSc119.2 sites were detected within the D haplome (Figs. 3 and 5f; Table 2).

#### Chromosomal alterations – overview

Seven types of structural alterations and one numerical alteration occurred in the three *E.* ×*mucronata* cytotypes. The following structural alterations were found (Table 3). (1) H/St translocation of a *Pseudoroegneria*-derived centromeric chromosomal segment to a *Hordeum* chromosome, which occurred in all the analysed plants (twice in C9, 50–1 and 50–7) (Figs. 2a, c, 4a, c and 5a, c and e) and was the only type out of the seven that has also been detected in either parental species of *E.* ×*mucronata* (the translocation was detected on a pair of chromosomes in *E. repens* [18]). Thus, this translocation is supposed to be inherited from a parent (see Discussion). The translocated chromosome carried a pSc119.2 site in the terminal region of one chromosome arm (when this probe was used). (2) H/St translocation, in which a *Pseudoroegneria-*labelled chromosome carried a *Hordeum* probe signal in its centromeric region, and this translocation only occurred in the nonaploid 41–5 (Fig. 5e). (3) H/St translocation occurred between terminal parts of the *Hordeum* and *Pseudoroegneria* chromosomes. Since the translocation caused acquisition of the co-localised 18S–5S–18S rDNA site by a *Pseudoroegneria* chromosome (Figs. 3 and 4) and disappearance of this site in *Hordeum*, we classified this translocation as reciprocal, and it occurred in both heptaploids (Fig. 4a and c). (4) H/St terminal translocation occurred when a *Pseudoroegneria*-like chromosome carried a terminal segment translocated from a *Hordeum*-like chromosome. This type of translocation occurred only in the heptaploids on two chromosomes (Fig. 4a and c). One of the chromosomes carried a co-localised 5S–18S rDNA site adjacent to the breakpoint and a pSc119.2 site in the terminal region of the opposite chromosome arm (pSc119.2 was applied only in C9). (5) D/H reciprocal translocation of a chromosome segment occurred between *Hordeum-* and *Aegilops*-like chromosomes. The breakpoints on both involved chromosomes were close to an 18S rDNA site, and the translocated segment of the *Aegilops* chromosome included a pSc119.2 site, and it was specific to the nonaploid 50–1 (Figs. 3 and 5a). (6) D/V interstitial translocation is a non-reciprocal translocation of a chromosomal segment from an *Aegilops*-like chromosome to a *Dasypyrum*-like chromosome. The translocated chromosome carried two pSc119.2 sites (in interstitial and subtelomeric positions) and was found in both heptaploid plants (Fig. 4a and c). (7) Inversion within a *Hordeum*-labelled chromosome, where the co-localised 5S–18S rDNA locus was in a mutually inverted position in nonaploid plants 50–1, 50–7 in comparison to 41–5 (the rDNA locus occurred on the same and opposite chromosome arms, respectively; Figs. 3 and 5b, d and f).

**Table 3:** Characterisation and occurrence of structural chromosomal alterations in the analysed plants and parental species

No.	Type of chromosomal alteration	Involved haplomes	Chromosomal segment	10–1	17–4	C9	C25B	41–5	50–1	50–7	*E. repens*	*E. intermedia*
1	translocation	H/St	centromeric	1	1	2	1	1	2	2	2	–
2	translocation	H/St	centromeric	–	–	–	–	1	–	–	–	–
3	translocation	H/St	terminal	–	–	1	1	–	–	–	–	–
4	translocation	H/St	terminal	–	–	2	2	–	–	–	–	–
5	translocation	D/H	terminal	–	–	–	–	–	1	–	–	–
6	translocation	D/V	interstitial	–	–	1	1	–	–	–	–	–
7	inversion (pericentric)	H	interstitial	–	–	–	–	–	1	1	–	–

A numerical chromosomal alteration was observed in one heptaploid plant (C9). Although the total number of chromosomes in this plant was euploid, not all chromosome sets exhibited multiples of seven chromosomes. One missing chromosome from the *Pseudoroegneria* chromosome set was compensated by an extra chromosome from *Hordeum*. The identity of the extra and missing chromosomes was traceable based on comparison with the second heptaploid; the extra *Hordeum* chromosome was one of the two chromosomes with the translocated centromeric region from *Pseudoroegneria*, although one *Pseudoroegneria*-like chromosome carrying a co-localised locus for 5S and 18S rDNA was missing (Fig. 4c and d; Table 3).

## Discussion

The occurrence of natural hybrids with high ploidy levels has rarely been documented for hybrid complexes from the *Triticeae* tribe*.* For example, there are reports of a heptaploid hybrid between *Thinopyrum junceum* and *Elytrigia repens* from Sweden [30] and a nonaploid hybrid of *Elytrigia pycnantha* and *E. repens* from France [31]. GISH analysis of the latter hybrid revealed the presence of four haplomes within this nonaploid, which consisted of four chromosome sets from *Pseudoroegneria*, two chromosome sets from *Agropyron*, two chromosome sets from *Thinopyrum* and one chromosome set from *Hordeum*. These results demonstrate that regular meiosis in higher polyploids may occur in hybrid complexes within *Triticeae*. However, to the best of our knowledge, the presence of four haplomes has not been shown in hexaploid and heptaploid natural hybrids.

In this study, we show that *Elytrigia ×mucronata* is an allopolyploid of high complexity, in which all three studied hybrid cytotypes comprised all four haplomes (D, H, St, V) present in the parental species *E. repens* and *E. intermedia*. In all but one case (see below), all of the chromosome sets in the hybrids were euploid and consisted of seven chromosomes. While the genomic constitution (i.e., the type and number of specific haplomes/basic genomes) of the *E. ×mucronata* hybrids reflects the ploidy level of particular cytotypes, it also depends on the type of gametes involved in the origination of particular plants.

If we assume that regular meiosis occurs in the parental species, then the *E.* ×*mucronata* hexaploid would harbour three chromosome sets from *Pseudoroegneria* and one chromosome set each from *Hordeum, Aegilops* and *Dasypyrum* (genomic formula StStStHDV). In addition to these chromosome sets, the heptaploid cytotype harboured an additional chromosome set from *Pseudoroegneria* (StStStStHDV). The genomic constitution of the nonaploid cytotypes differed between plants. Two plants exhibited 35 St + 14 H + 7 D + 7 V chromosomes, while the other nonaploid harboured 28 St + 7 H + 14 D + 14 V chromosomes. We assume that this difference reflects the distinct origins of the nonaploid cytotypes [16] (see below).

### Origin of different cytotypes

**Fig. 6:**
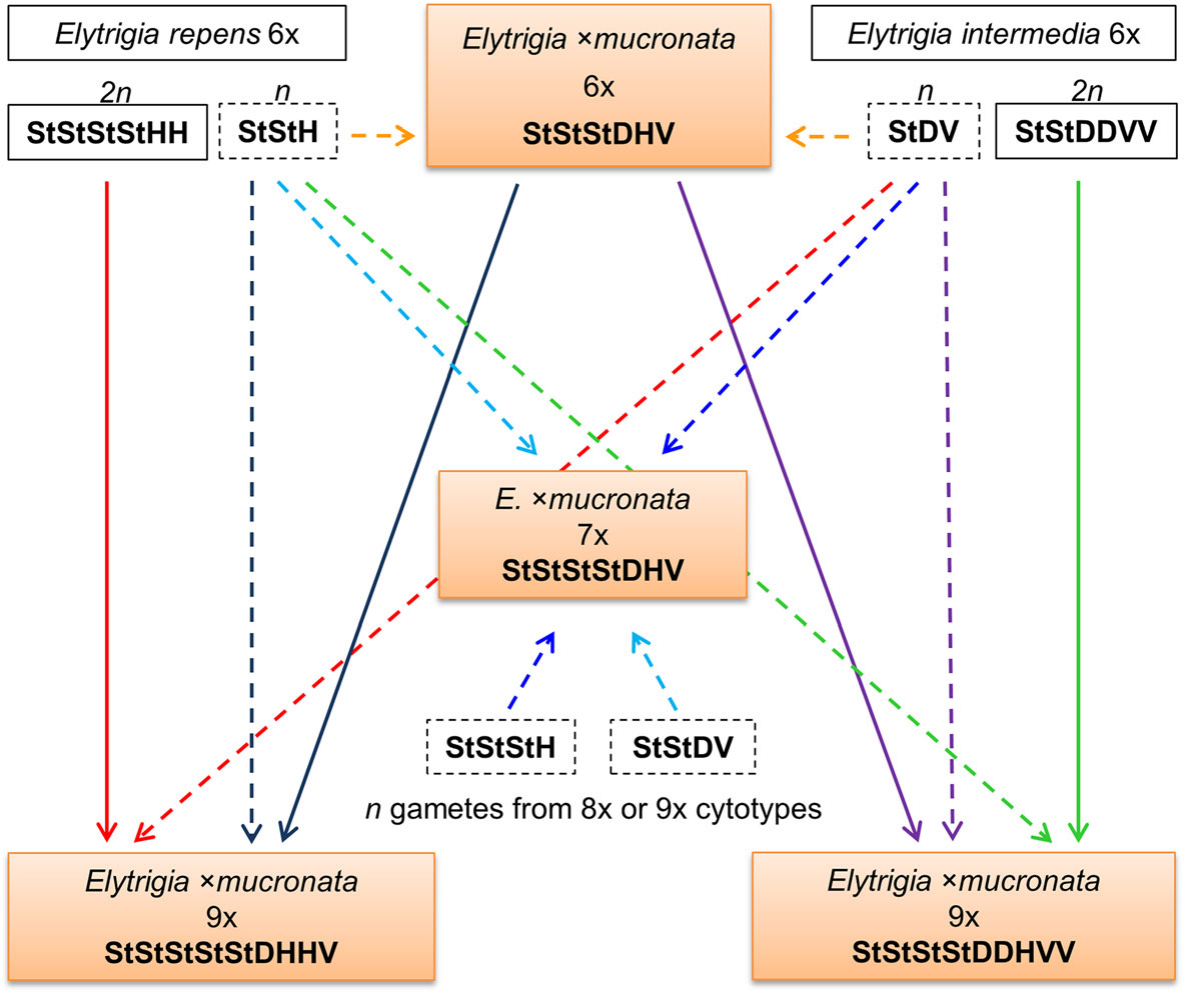
Proposed scenarios of the origination of *E.* ×*mucronata* cytotypes. The analysed cytotypes (6x, 7x, 9x) are presented in coloured boxes; for each cytotype, the genomic formula is given. For parental species, the contributions of unreduced (2n, solid boxes) and reduced (n, dashed boxes) gametes are considered. The contributions of unreduced and reduced gametes are indicated with solid and dashed arrows, respectively. Arrows of the same colour represent one potential scenario

The genomic constitutions of the analysed plants allowed us to partly infer the types of gametes that gave rise to their origination (Fig. 6). The hexaploid cytotype of *E.* ×*mucronata* most likely originated through the merging of two reduced gametes from both parental species.

As mentioned above, nonaploid plants most likely originated through the fusion of reduced and unreduced gametes [15, 16]. Mahelka et al. [16] suggested different scenarios for the origin of the hybrid nonaploids 50–1, 50–7 and 41–5 (plants N7, N6 and N8 in the original article). While plants 50–1 and 50–7 may have arisen from 2n (*E. repens*) + n (*E. intermedia*) or 2n (6x *E*. ×*mucronata*) + n (*E. repens*) combinations, nonaploid 41–5 may represent either 2n (*E. intermedia*) + n (*E. repens*) or 2n (6x *E. * ×*mucronata*) + n (*E. intermedia*) gamete compositions. Since the alternative gamete combinations result in the same genomic compositions, we are not able to discern which scenario truly led to the formation of the analysed nonaploids by using GISH. The involvement of hexaploid *E.* ×*mucronata* hybrids in the formation of the nonaploids seems to be more likely because hybrids might more easily produce unreduced gametes than pure species due to disturbed meiosis [9]. The heptaploid cytotype likely resulted from heteroploid hybridisation; however, the exact mode of its origination is difficult to determine. One possibility is that the heptaploid originated after a cross between a hexaploid and an octoploid (2n = 8x = 56). If the hexaploid parent was either *E. repens* or *E. intermedia* (gamete *n* = 3x = 21 = StStH or StDV, respectively), then the gamete from the octoploid would have to have been *n* = 4x = 28 = StStDV or StStStH, respectively (Fig. 6). However, no octoploid plants have been recorded from this locality. Alternatively, the heptaploids could have originated after a cross between a hexaploid and a nonaploid, in which the latter was the donor of the gamete comprising four chromosome sets. Such a scenario has been observed in *Elytrigia* wheatgrasses, where a heptaploid plant was found among progeny of the nonaploid hybrid 50–1 collected in the field [16]. It is likely that the pollen donor was either *E. repens* or *E. intermedia*. Unfortunately, the genomic constitution of this particular heptaploid was not analysed.

### Chromosomal alterations in *E.* ×*mucronata*

Chromosomal alterations occurred in all three cytotypes and involved all four haplomes. Most of the translocations involved St and H chromosomes, while the V chromosome from *Dasypyrum* was involved in only one translocation. The question is whether this difference simply occurred because St and H chromosomes outnumber chromosomes from the other haplomes, or if it stems from different levels of karyotype stability, which may have a strong effect on chromosome restructuring and aneuploidy in *Triticeae* [32].

Structural chromosomal alterations have been reported in other perennial species harbouring an H and/or St haplome. Dou et al. [33] found two types of non-reciprocal translocations between H and St haplomes and two types of reciprocal translocations between H and Y haplomes in *Elymus nutans* (2n = 6x = 42, StStHHYY). Different frequencies of chromosomal alterations between particular haplomes were observed in *Kengyilia thoroldiana* (2n = 6x = 42, StStPPYY). The frequency of P/Y translocations was higher than that of P/St translocations, while no translocations were observed between the chromosomes of the St and Y haplomes [34].

The question arises of how frequently and at which stage of hybrid formation do translocations occur? Without knowledge on the parental species, it is problematic to infer whether particular structural rearrangements have been inherited from the parental species, or whether they have originated de novo in hybrids. Cytogenetic analyses of local accessions of parental species *E. intermedia* and *E. repens* showed that in *E. repens*, one pair of *Hordeum* chromosomes carried a centromeric H/St ‘translocation’ [18], corresponding to what we called Type 1 translocation here. In contrast, no translocation that would resemble those observed in the *E.* ×*mucronata* plants analysed here were observed in the other parental species *E. intermedia* [19, 23]. Therefore, we concluded that all but one (type 1) chromosomal alterations in the three cytotypes of *E.* ×*mucronata* appeared during hybrids’ formation.

In any case, the presence of only one such translocation in the hexaploid hybrid indicates the occurrence of regular meiosis in the *E. repens* parent. Thus, it appears that no de novo translocations appeared in the *E. ×mucronata* hexaploid. The presence of two such translocations in two nonaploids (50–1 and 50–7) and one translocation in the other nonaploid (41–5) is consistent with both alternative origins of these nonaploids.

Furthermore, the presence of the D/H reciprocal translocation (type 5) in the nonaploid 50–1 indicates that this plant is not a primary nonaploid hybrid between *E. intermedia* and *E. repens*, since recombination between H and D haplomes (which do not co-exist in any of the parents) must have occurred in the hybrid plant. Therefore, an origin involving a 2n (6x *E*. ×*mucronata*) + n (*E. repens*) combination seems more likely in this plant.

The notably higher frequency of chromosomal alterations observed in heptaploid plants contrasted with the results for the other analysed cytotypes. Three of the four types of translocations (types 3, 4, and 6) were not found in other cytotypes, suggesting that several multivalents between homoeologous chromosomes must have occurred during the formation of the gametes, giving rise to the heptaploid plants.

The concurrent presence of both chromosomes with reciprocally translocated segments indicates alternate segregation (i.e., the translocated chromosomes do not segregate and are present in a single gamete). It is unlikely that this state originated from the fusion of two unbalanced gametes. Non-reciprocal translocations can be non-reciprocal per se or may result from adjacent segregation, where normal and translocated chromosomes segregate into one gamete [35].

Moreover, the numerical chromosomal alteration observed in one heptaploid plant was an example of hidden aneuploidy [27, 32], i.e., the absence of one chromosome is compensated by the acquisition of an extra chromosome from another chromosome set.

Chromosomes were also observed carrying differentiating signals in the centromeric regions in *Dasypyrum*-like chromosomes. This feature has previously been reported for *E. intermedia* species [19, 23]. Further research is required to reveal the true nature of these GISH patterns and determine whether they resulted from chromosome restructuring or sequence homology.

### Mapping of repetitive DNA in *E*. ×*mucronata* hybrids

The evolution of ribosomal DNA genes in relation to allopolyploidy is an intensively studied issue (e.g., [36, 37]). rDNA loci are valuable chromosome markers, and the mapping of rDNA loci using in situ hybridisation allows for the evaluation of the progenitor-derivative patterns and positional dynamics of ribosomal loci within allopolyploid genomes. rDNA genes in allopolyploid species may experience contrasting and barely predictable patterns of evolution, ranging from loss of some loci with respect to their progenitors (*Zingeria*—[38]) over nearly complete additivity (e.g., *Thinopyrum ponticum*—[39]; *Nicotiana*—[40]) to increasing the number of rDNA loci (*Triticum*—[41]). *Triticeae* grasses are characterised by the ability to change the positions of rDNA loci [42–44], which may occur via the dispersion of minor loci, followed by rDNA array magnification and deletion of the original loci thereafter. Loss of rDNA loci is one potential mechanism accelerating the process of concerted evolution [45].

In all investigated hybrid cytotypes, the 5S and 18S rDNA loci were located on all chromosome sets representing the different haplomes. Therefore, the rDNAs of the *E*. ×*mucronata* hybrid reflect the principle of genome additivity to a certain degree. However, because *E*. ×*mucronata* is a hybrid between two allopolyploid species, the dynamics of the rDNA loci of this hybrid are traceable, provided that the pattern in both parental species is understood. Thus, we characterised specimens of both *E. repens* and *E. intermedia* from the same distribution area from which the hybrids originated ([18, 23], Mahelka, Kopecký, unpubl. data). In both parental species, we encountered some reorganisation of rDNA loci with respect to their diploid progenitors, which likely occurred following the origination of the allopolyploids (for discussion, see [18, 23]). The patterns of the rDNA loci of both *E. repens* and *E. intermedia* are shown in Additional file 1: Table S1. Notably, severe elimination of all but one minor 45S rDNA locus likely occurred within the *Hordeum* subgenome in *E. repens* (genomic formula StStStStHH). Similarly, the elimination of some 45S rDNA loci occurred within the *Pseudoroegneria*- and *Dasypyrum*-like subgenomes in *E. intermedia* (genomic formula StStDDVV). In both species, 5S rDNA was less heavily affected by the loss of the loci than 45S rDNA.

In this study, we predicted the theoretical numbers of rDNA loci in hybrid *E*. ×*mucronata* cytotypes (Additional file 1: Table S1) by assuming complete additivity of the parental rDNA loci. From comparison of the observed data, we can infer the positional dynamics of rDNA loci in the hybrids. For this purpose, we consider the 18S and 45S probes to be equally informative.

The total numbers of 5S rDNA loci fell well into the expected numbers in all hybrid plants examined. In the nonaploid 41–5, the number of 5S rDNA loci only fell within the expected range if the [2n (6x *E*. ×*mucronata*) + n (*E. intermedia*)] scenario of its origin was considered. Under the opposite scenario [2n (*E. intermedia*) + n (*E. repens*)], a lower number was observed (11 vs. 13–15 expected). This depletion was mainly caused by a smaller number of 5S rDNA sites on *Pseudoroegneria* chromosomes than expected (4 sites observed vs. 6–7 expected). However, we must note that the real number of loci in this plant may be higher than was observed because one chromosome was missing in the analysed metaphases. Such an observation is otherwise in agreement with the pattern found for 5S rDNA loci in the parental species, in which no major changes in the 5S rDNA loci were recorded ([23], unpubl. data).

In contrast, the total numbers of 18S rDNA sites were always higher than expected in the hybrids of all analysed cytotypes. This observation is especially interesting if we consider that severe losses of 45S rDNA loci had already occurred in both parental species [18, 23]. Restoration of some loci clearly occurred within the *Hordeum* and, to a lesser degree, *Pseudoroegneria* haplomes (see hexaploid 10–1) after the hybridisation events. In particular, while examining the *Hordeum* haplome, we observed co-localised 5S–18S and/or 18S–5S–18S rDNA loci in all the cytotypes (although in both heptaploids, the co-localised locus had been translocated to a *Pseudoroegneria* chromosome); however, this locus was not observed in *E. repens* [18]. This pattern was consistent in all three cytotypes, but neither the mechanism of the re-appearance of the loci nor its cause was studied.

We did not probe either of the parental species with the pSc119.2 probe. In other studies, up to 5 chromosomes with one or two pSc119.2 loci (located in a terminal or interstitial position) have been found in *E. intermedia* [46]. In *E. repens,* the total number of pSc119.2 sites ranges between 5 and 10 (one interstitial site and others in the terminal region) [47]. Although these plants come from different geographic regions, it appears that similar to 18S rDNA, the total number of pSc119.2 sites detected in *E. ×mucronata* was higher than that in the parental species.

## Conclusions

Hybridisation and polyploidisation are prominent speciation mechanisms in the grass tribe *Triticeae*. These mechanisms have not only produced new allopolyploid lineages, but the ongoing transfer of genetic material via extensive hybridisation and introgression may also have significantly enriched the gene pools of the newly established lineages, thus providing raw material for selection. Many *Triticeae* wheatgrasses, including *E. intermedia*, represent invaluable in situ sources of genetic material that may be useful for wheat improvement. Therefore, the newly established hybrid lineages are both of interest to general science and of particular concern to wheat breeders due to their potential practical impact. We performed molecular cytogenetic analyses using genomic and fluorescence in situ hybridisation on three cytotypes of the allopolyploid *E.* ×*mucronata*, a hybrid between two allopolyploid wheatgrasses, *E. intermedia* and *E. repens*. In all three investigated cytotypes (i.e., hexaploid, heptaploid, and nonaploid), we observed coexistence of four different haplomes that occurred in the parental species, thus confirming the assumed hybrid origin of the plants. The genomic constitutions of the analysed plants allowed us to partially infer the types of gametes that gave rise to their origins. While the hexaploid cytotype of *E.* ×*mucronata* originated through the merging of two reduced gametes from both parental species, the heptaploid cytotype likely resulted from heteroploid hybridisation. The nonaploid plants most likely originated through the fusion of reduced (n) and unreduced (2n) gametes. The different genomic constitutions of the nonaploids showed that along with both parental species, the hexaploid *E.* ×*mucronata* should be considered as a donor of unreduced gametes. Several chromosomal alterations observed in both heptaploid and some nonaploid plants occurred during and/or after the formation of the hybrids. Moreover, a specific chromosomal translocation detected in one of the nonaploids indicated that it was not a primary hybrid. Therefore, at least some of the hybrids are fertile and produce viable offspring.

## Methods

### Plant material

The plant material used in this study is available as living material from previous studies [15, 16]. All investigated plants were collected by the author (VM) at localities where no permissions were necessary to collect the samples. Details on the sample locations are given in Table 1 and Fig. 1. The experiments were performed on selected hybrid plants and involved two hexaploid (2n = 6x = 42), two heptaploid (2n = 7x = 49), and three nonaploid (2n = 9x = 63) *E. ×mucronata* plants (Table 1). Hexaploid and nonaploid plants were used in previous studies focused on genome size variation and natural hybridisation [15, 16]. Thus, while hexaploid plants 10–1 and 17–4 correspond to accessions H-30 and H-2 from Mahelka et al. [16], nonaploid plants 41–5, 50–1 and 50–7 correspond to nonaploids N8, N7 and N6 from the same study, respectively. The heptaploids are studied for the first time in this study. The seeds of diploid *Triticeae* species used for probe preparation were provided by the US Department of Agriculture (USDA) National Small Grains Collection.

### Collecting materials and slide preparation

Plants were cultivated in plastic pots filled with perlite in the greenhouse. Root tips were pre-treated in ice cold water for 24–33 h and fixed in fresh ethanol-acetic acid fixative (3:1, v/v).

The fixed root tips were washed in distilled water (twice) and citrate buffer (0.01 M citric acid and 0.01 M sodium citrate buffer, pH 4.8) for 5 min each. Thereafter, the root tips were treated in an enzyme mixture [1% (w/v) pectinase, 1% (w/v) pectolyase and 20% (v/v) pectinase (Sigma, St. Louis, MO, USA) in citrate buffer] for 3–4 h at 37 °C. After treatment, the digested tissue was washed in distilled water.

The slides were prepared using the smear method [48] according to a previous report [49] with several modifications. The digested tissue was carefully transferred to a microscope slide, and a suspension was produced with needles. Cold 75% acetic acid was then immediately added to the suspension, after which the slide was placed on a hot plate and stirred with a needle to spread the cells. Finally, 150 μl of cold ethanol-acetic acid fixative was added, and the slides were washed with ethanol and air-dried.

### Probe preparation and in situ hybridisation

The following three probes representing repetitive chromosomal markers were used for the FISH analyses: (1) a 5S rDNA probe, (2) an 18S rDNA probe, and (3) a pSc119.2 probe. Ribosomal 5S and 18S DNAs were amplified using the primers 5SprobeF (5′-GATCCCATCAGAACTCCGAAG-3′), 5SprobeR (5′-CGGTGCTTTAGTGCTGGTATG-3′) [50], 18S-F (5′-CGAACTGTGAAACTGCGAATGGC-3′) and 18S-R (5′-TAGGAGCGACGGGCGGTGTGTG-3′) [51]. The pSc119.2 probe, a 120-bp fragment originally isolated from rye [52], was amplified from *Triticum aestivum* cv. Chinese Spring DNA according to the reported protocol.

The selection of the diploid species used to obtain the genomic probes was based on analyses of the genomic constitutions of the parental species [18, 19, 23]. Genomic DNAs from the diploid species *Aegilops tauschii* Coss. (USDA accession identifier PI542278; representing D haplome), *Dasypyrum villosum* (L.) P. Candargy (PI639751; V haplome), *Hordeum bogdanii* (PI269406; H haplome), and *Pseudoroegneria spicata* (Pursh) Á. Löve (PI563869; St haplome) were extracted using the DNeasy Plant Mini Kit (Qiagen, Hilden, Germany). The probes were labelled either via nick translation using the Biotin-Nick Translation Kit and the DIG-Nick Translation Kit (Roche, Indianapolis, IN, USA) according to the manufacturer’s protocol, or via random primer labelling [53] with direct fluorochromes (Cy3, Cy5; Amersham, Piscataway, NJ, USA).

The following three sequential in situ experiments were performed on mitotic metaphase chromosomes of *E. ×mucronata*: (i) FISH with the probes for 5S rDNA (Cy5), 18S rDNA (digoxigenin, anti-DIG-FITC conjugate), and pSc119.2 (Cy3, this probe was not applied in plants 17–4 and C25B); (ii) GISH with three genomic probes, from *Aegilops tauschii* (digoxigenin, anti-DIG-FITC conjugate), *Hordeum bogdanii* (biotin, streptavidin-Cy3), and *Pseudoroegneria spicata* (Cy5); and (iii) GISH with a genomic probe from *Dasypyrum villosum* (biotin, streptavidin-Cy3). The hybridisation pattern was always confirmed in 3–6 metaphases per plant.

Chromosomes and probes were simultaneously denatured at 93 °C for 3 min on a hot plate (ThermoBrite™ Slide Processing System, StatSpin, Norwood, MA, USA). Hybridisation was then performed at 63 °C for 3 h according to the relevant literature [54, 55]. Detection was completed using an anti-DIG-FITC conjugate (Roche, Indianapolis, IN, USA) or a streptavidin-Cy3 conjugate (Sigma, St. Louis, MO, USA) (1 h in 37 °C). Slides were mounted with Vectashield antifade mountant (Vector Laboratories, Burlingame, CA, USA), examined and photographed on a Zeiss Axio Imager.Z2 microscope system equipped with an ApoTome.2. Zen (Zeiss, Jena, Germany) and Adobe Photoshop software (Adobe Systems, San Jose, CA, USA) were used for merging and processing the images.

## Additional file

Additional file 1: **Table S1.** Theoretical patterns of rDNA loci in hexaploid (2n = 6x), heptaploid (2n = 7x), and nonaploid (2n = 9x) cytotypes of *E.* ×*mucronata* hybrids. Observed values of rDNA loci for the parental species and expected values for the investigated *E.* ×*mucronata* cytotypes. (XLSX 12 kb)

## Abbreviations

18S rDNA: 18S ribosomal DNA
45S rDNA: 45S ribosomal DNA
5S rDNA: 5S ribosomal DNA
anti-DIG-FITC: Anti-digoxigenin-fluorescein isothiocyanate
Cy3: Cy3-dUTP, Cyanine 3-dUTP
Cy5: Cy5-dUTP, Cyanine 5-dUTP
DAPI: 4′,6-Diamidino-2-phenylindole
DNA: Deoxyribonucleic acid
FISH: Fluorescence in situ hybridisation
GISH: Genomic in situ hybridisation
ITS: Internal transcribed spacer
rDNA: Ribosomal DNA
streptavidin-Cy3: Streptavidin-cyanine 3
